# Exome Analysis Identified Novel Homozygous Splice Site Donor Alteration in *NT5C2* Gene in a Saudi Family Associated With Spastic Diplegia Cerebral Palsy, Developmental Delay, and Intellectual Disability

**DOI:** 10.3389/fgene.2020.00014

**Published:** 2020-02-21

**Authors:** Muhammad Imran Naseer, Angham Abdulrahman Abdulkareem, Peter Natesan Pushparaj, Fehmida Bibi, Adeel G. Chaudhary

**Affiliations:** ^1^ Center of Excellence in Genomic Medicine Research, King Abdulaziz University, Jeddah, Saudi Arabia; ^2^ Department of Medical Laboratory Technology, Faculty of Applied Medical Sciences, King Abdulaziz University, Jeddah, Saudi Arabia; ^3^ Special Infectious Agents Unit, King Fahd Medical Research Centre, King Abdulaziz University, Jeddah, Saudi Arabia; ^4^ Center for Innovation in Personalized Medicine, King Abdulaziz University, Jeddah, Saudi Arabia

**Keywords:** *NT5C2* gene, splice site mutation, developmental delay, microcephaly, spastic diplegia

## Abstract

Hereditary spastic paraplegias (HSPs) is a rare heterogeneous group of neurodegenerative diseases, with upper and lower limb spasticity motor neuron disintegration leading to paraplegias. *NT5C2* gene (OMIM: 600417) encode a hydrolase enzyme 5'-nucleotidase, cytosolic II play an important role in maintaining the balance of purine nucleotides and free nucleobases in the spinal cord and brain. In this study we have identified a large consanguineous Saudi family segregating a novel homozygous splice site donor alteration in *NT5C2* gene leading to spastic diplegia cerebral palsy, developmental delay and microcephaly. Whole exome sequencing (WES) was performed for the affected members of the family to study the novel mutation. WES data analysis, confirmed by Sanger sequencing analysis, identifies a homozygous splice site donor alteration of possible interest in *NT5C2* (ENST00000343289: c.539+1G > T) at the sixth exon/intron boundaries. The mutation was further ruled out in 100 healthy control from normal population. The novel homozygous mutation observed in this study has not been reported in the literature or variant databases. The identified splicing alteration broadens the mutation spectrum of *NT5C2* gene in neurodevelopmental disorders. To the best of our knowledge this is the first report from Saudi Arabia.

## Introduction

Hereditary spastic paraplegias (HSPs) is characterized as nonprogressive motor deficits as a result of cerebral abnormalities arise in the prenatal or perinatal stage. Signs and symptoms will found at early stage of life as result of loss of corticospinal motor function (Blackstone, 2012). Recently, it has been reported that the HSPs exist not only in “pure” forms but also in “complex” forms that are linked with other neurologic and extra neurologic conditions. Moreover, the HSPs are among the most genetically diverse neurologic disorders, with well over 70 distinct genetic loci, for which about 60 mutated genes have already been identified (Blackstone, 2018). The diagnosis of HSP is based on the presence of a clinical history of progressive spastic paraparesis in a pure form or associated with other neurological and systemic manifestations (with or without familial history), evidence of a genetic mutation in a *locus* related to a phenotype of HSP previously described in the literature, and exclusion of structural disorders or other genetic or acquired conditions that explain the clinical picture (Sedel et al., 2007; De Bot et al., 2010; Lo Giudice et al., 2014; Klebe et al., 2015; Pedroso et al., 2015; Souza et al., 2015). Generally, the clinical picture of an HSP is characterized by a subtle onset and slowly progressive course of spastic paraparesis described by patients as abnormal gait, leg stiffness, or gait instability. It is very difficult to HSP because the other neurological and systemic features that characterize complicated forms may precede the onset of spastic paraparesis (Harding, 1993; Sedel et al., 2007; Salinas et al., 2008; De Bot et al., 2010; Pedroso et al., 2015).

Cerebral palsy (CP) is characterized by large group of impairing disorders control of movement due to lesion or defect in the developing brain. This disease is common in childhood (Hughes and Newton, 1992), with prevalence of about 1 in 250 to 1,000 births (Pharoah et al., 1987). People with CP might have an issue with quality motor abilities or maintaining stability and walking, or have involuntary actions, including uncontainable motions of the hands or slobbering. CP is the most common severe motor disability in children, and its severity is demonstrated by the fact that 40% of children with the condition cannot walk independently (Korzeniewski et al., 2018; Christensen et al., 2014). In some cases patients have epilepsy and intellectual disability, and in some cases newborn with CP having abnormally small head size (microcephaly). This disorder can be split into four essential categories conferring to the movement disturbance: spastic, ataxic, athetoid, and mixed forms (Ninds, 2005). About 70–80% of cases are spastic CP, and it is subdivided into monoplegic, quadriplegic, diplegic, and hemiplegic types, depending on limbs that are affected. CP patients normally show increased hypertonia, deep tendon reflexes, and muscle weakness along with scissors gait as common feature. Spastic quadriplegia is the most severe form of spastic CP if accompanied by dysarthria. The most common mixed forms are spastic and athetoid movements cover 10–20% of total number of cases, but some other combinations may also possible (Rajab et al., 2006).

Genetic forms of CP cover around 2% of the population in Europe, and they are supposed to be caused by consanguineous marriage within families (Mitchell and Bundey, 1997). Also, any variation in genetic defects in nucleotide metabolism-related genes leads to the genetic forms of CP (Novarino et al., 2014). Microdeletions or microduplications have been reported in patients with diagnoses of cerebral palsy. Previous studies have reported between 10 and 12% detection rate of likely pathogenic CNV in patients with cerebral palsy. The genes included in these CNVs include *KANK1*, *WDR45*, *HSPA4*, and *SPAST* (Fahey et al., 2017). *NT5C2* genes encode the 5'-nucleotidase, cytosolic II, protein, which is involved in the repairs of intracellular nucleotides composition of purine/pyrimidine in support with other nucleotidases. In addition, this enzyme has an important role in maintaining the stability of nucleotides, nucleosides, and free nucleobases of purine in the central nervous system. The *NT5C2* gene catalyzes the adenosine hydrolysis along with monophosphate and inosine monophosphate-releasing adenosine which play a key role in promoting myelin formation in the central nervous system. Adenosine prevents proliferation of oligodendrocyte progenitor cells, stimulates their differentiation into mature oligodendrocytes, and controls the communication of neurons and glial cells with the help of axons (Elsaid et al., 2017).

In this study, we identified a homozygous splice site donor alteration in *NT5C2*. The homozygous mutation causes a loss of function mutations in this gene including other splice site donor mutations that have been linked to an autosomal recessive form of spastic paraplegia. Common features among patients include spasticity, gait abnormalities, speech delay, brain abnormalities, and ophthalmological signs. A single patient was also noted to have microcephaly (Novarino et al., 2014).

### Case Report

#### Electroencephalography Report

**Figure 1:**
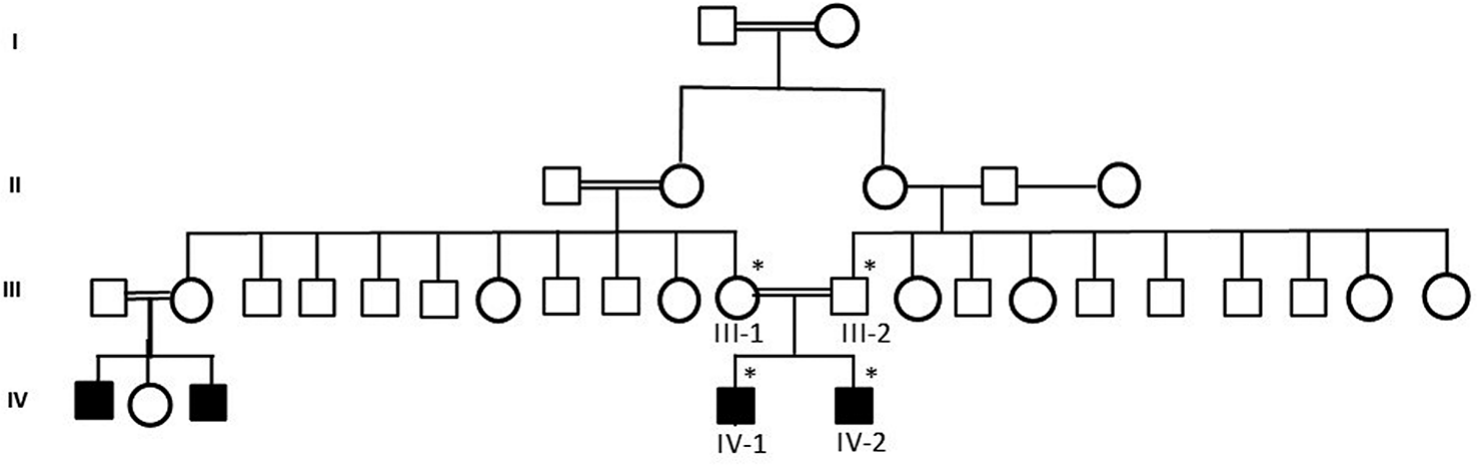
A pedigree of a consanguineous Saudi family. The available samples are marked (*) as satiric symbol.

A sleep EEG record revealed a background of predominately beta waves. Paroxysmal epileptic discharge was noted in both (IV-1 and IV-2) the affected individuals as shown in the pedigree **Figure 1**, primarily in the right central hemisphere proband.

### Whole Exome Sequencing

The obtained variant call format (VCF) file contains 86,390 variants. We used different filter based on quality, frequency, genomic position, protein effect, pathogenicity, and based on previous known associations with the phenotype. Subsequently, homozygous gene variants were considered. Homozygous splice site donor alteration was of possible interest in the *NT5C2* at the sixth exon/intron boundaries (ENST00000343289: c.539+1G > T) intron. WES results showed pathogenic mutation in the *NT5C2* gene where G at position 539 is substituted by T in the intronic region, resulting in change of amino acid glycine residue into a tyrosin. Thus, the novel homozygous mutation in *NT5C2* gene was found only in the affected members of the family.

### Sanger Sequencing

**Figure 2:**
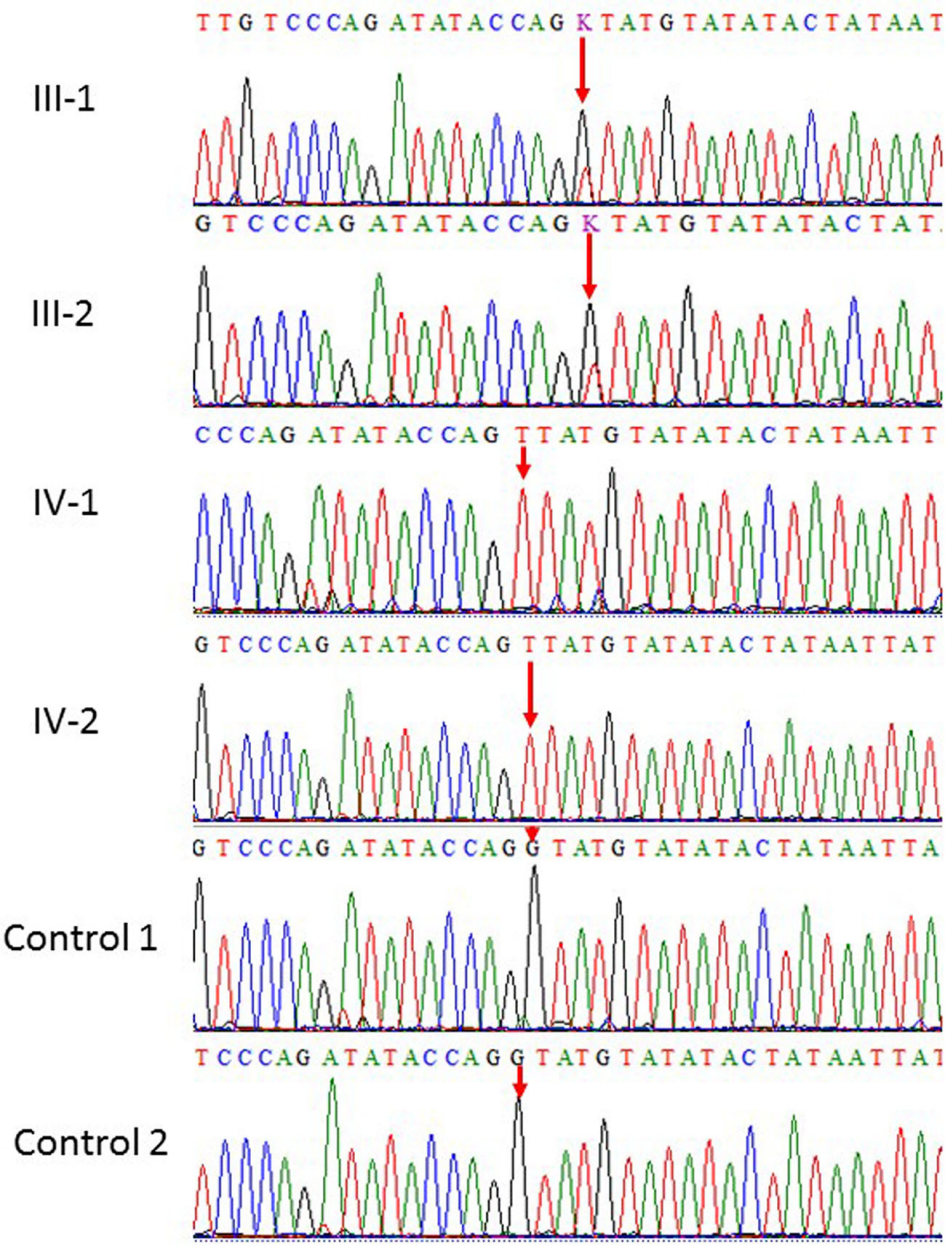
Sanger sequence analysis: a and b (III-1 and III-2) are normal parents and normal female, while (IV-1 and IV-2) are affected children showing only homozygous where G at position 539 replaced by T in the intronic region resultantly amino acid glycine residue into a tyrosin in *NT5C2*.

Our results showed homozygous splice site donor alteration of possible interest in NT5C2 gene (ENST00000343289: c.539+1G > T) in both affected subjects IV-1 and IV-2 proband, whereas the parents III-1 and III-2 are heterozygous at this position. The change of bases results in a replacement of a conserved glycine residue into a tyrosine in the intron of the *NT5C2* gene. The mutation spectrum in *NT5C2* gene known so far is mentioned in the **Table 1**. None of the 100 unrelated healthy samples has this sequence variation. The both parents were heterozygous, which also confirms the mode of inheritance, as shown in **Figure 2**.

**Table 1:** Mutation spectrum in *NT5C2* gene known so far.

S. No	Mutation	Consequence	State	Origin	Reference
1	c.1379T > C	p.Leu460Pro	Homozygous	Arab Muslim origin	9
2	c.1159 + 1G > T		Homozygous	Qatari	6
3	c.86G-A	Arg 29 ter	Homozygous	2 Turkish sibs	5, 8
4	c.1225delA	Ser409Valfs436Ter	Homozygous		5
5	c.988-1G-T	Splice	Homozygous		5
6	c.445A-T	arg149-to-ter	Homozygosity		5
7	c.175+1G-A	p. Ala64Val	Homozygous		5
**8**	**c.539+1G > T**		**Homozygous**	**Saudi Arabia**	**Present study**

## Discussion

The NT5C2-related phenotype has earlier been clinically assigned to CP (MIM: 613162) and consanguineous marriage within the family we described has an important role in a genetic form of spastic CP (Bundey and Griffiths, 1977). This defect is caused by a homozygous splice site donor alteration of possible interest in gene NT5C2 (ENST00000343289: c.539+1G > T).

Dursun et al, 2009 reported five Turkish siblings suffering from CP with mental retardation. Novarino *et al*. identified a homozygous c.86G > A transition in the NT5C2 gene, resulting in p.R29X substitution. Two affected sisters from a consanguineous family segregating by spastic paraplegia-45 was identified by homozygosity for a 1-bp deletion (c.1225delA) in the NT5C2 gene, resulting in a frameshift and premature termination (Ser409Valfs436Ter). None of the unaffected family members were homozygous for this mutation. Novarino *et al*. also found homozygosity for an acceptor splice site mutation (c.988-1G > T) in the *NT5C2* gene in two affected sisters from a consanguineous family. None of the unaffected family members were homozygous for this mutation. In the same study, they reported a homozygosity caused by c.445A > T transversion in the *NT5C2* gene, resulting in p.Arg149Ter substitution. In addition, homozygosity for a donor splice site mutation (c.175+1G-A) was identified in two affected siblings (Novarino et al., 2014). Recently Elsaid et al. reported a consanguineous Qatari family having a novel homozygous c.1159+1G > T in *NT5C2* splice site mutation (NM_012229.4/NM_001134373.2). Among all other patients reported so far with CP, they were unique in that they displayed persistent early in 2017,truncal hypotonia, dysarthria, and variable-sized patches of brownish skin staining at early stage of life (Elsaid et al., 2017). Straussberg describes the clinical, radiologic, and genetic findings in three affected members of the consanguineous family from Arab region CP belonging to that harbors a novel mutation in *NT5C2* (c.1379T > C; p.Leu460Pro) (Elsaid et al., 2017). Moreover, recently exon rearrangement c.771 + 573_814-298del reported in the in *NT5C2* loci in a family with a complex genetic hereditary spastic paraplegias (Darvish et al., 2017).

## Conclusions

In conclusion, the genetic form of HSPs tends to be rare, but our report, suggests that consanguineous marriage plays an important role in the genetic form of spastic CP with intellectual disability and microcephaly. We reporting for the first time from Saudi Arabia a novel homozygous splice site donor alteration of possible interest in *NT5C2* c.539+1G > T further explain the genetics involved in such type of disorders will provide an opportunity to understand the fundamental causes of CP in the future.

## Materials and Method

### Ethical Approval and Sample Collection

Samples from a consanguineous Saudi family were obtained by following appropriate local ethical approval protocols and guidelines from the King Abdulaziz University Hospital, Jeddah. Informed consent was taken from all the member of the family participated in this study according to the Declaration of Helsinki. To maintain the anonymity of the family, the datasets in this paper will not be publicly deposited. Requests to access the datasets should be directed to corresponding author. The study was done after the approval by the ethical committee of the Center of Excellence in Genomic Medicine Research, King Abdulaziz University, Jeddah. Magnetic resonance imaging (MRI) and electroencephalography (EEG) were performed to exclude any infection or trauma. DNA was extracted from blood samples by using the MegNA Pure 24 system from Roche Life Science. Detailed disease history was taken and pedigree was drawn using information from the family. The samples were prepared for exome sequencing after checking the quality and concentration according to the Agilent Sure Select Target Enrichment Kit preparation guide. The blood samples were collected from all 4 members of the family (2 affected children and 2 parents) and 100 healthy people of Saudi origin as control group as shown in pedigree (**Figure 1**). Affected individuals were under treatment and medical examination at King Abdulaziz University Hospital, Jeddah.

#### Patient 1

**Figure 3:**
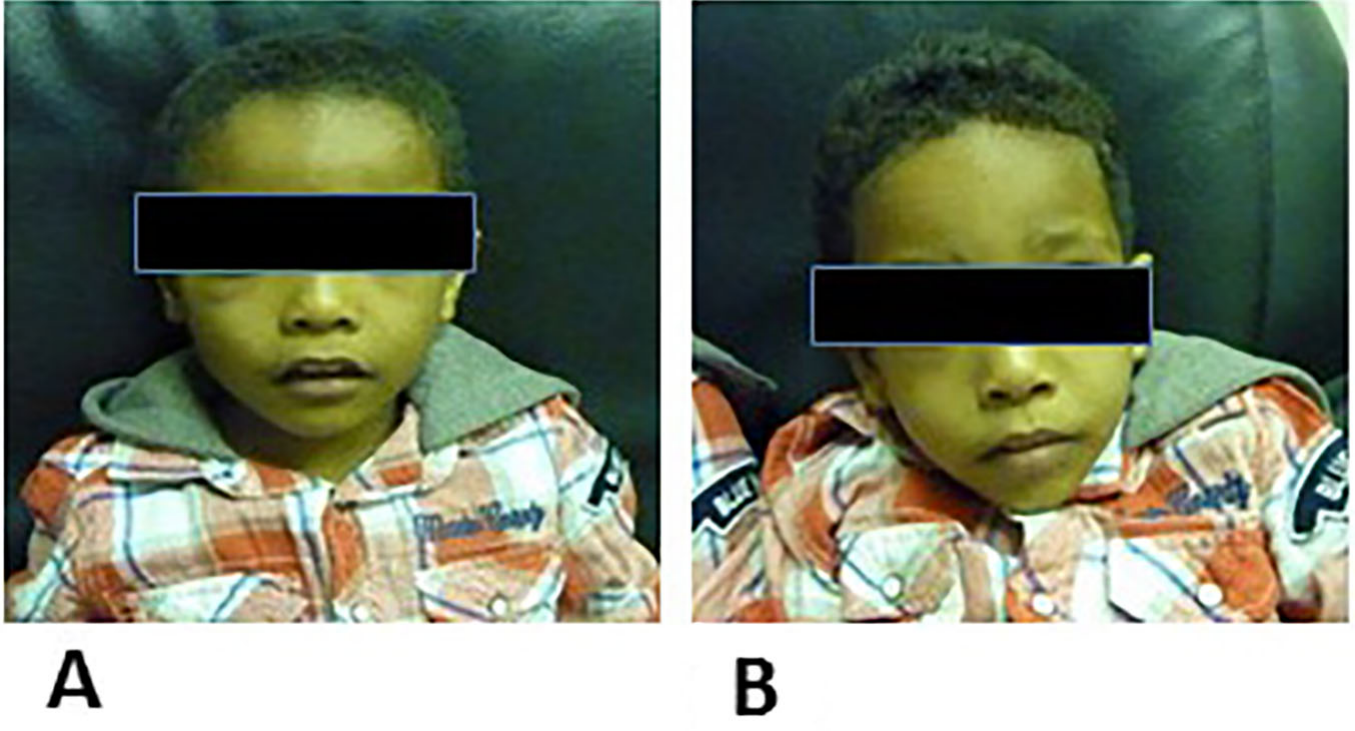
**(A)** is the facial apperance of proband IV-1 and **(B)** is the facial apperance of poband IV-2.

Patient 1 (IV-1) is 5 years old, and he was born by normal vaginal delivery at full term without any complication. He has one brother and two relatives affected with the same problem. Clinical examination showed that he generally looks well, his head circumference is 45 cm, and his central nervous system shows hypertonia in the lower limbs and brisk deep tendon reflexes. He started speaking and walking at age 2. The CT scan of his brain showed density in the left frontal cerebrospinal fluid, an extra axial lesion, and a left frontal subarachnoid epidermal cyst. His thyroid profile was normal, as was a complete blood count. A sleep EEG record revealed a background of predominately beta waves. Paroxysmal epileptic discharge was noted in IV-1 the affected individuals, primarily in the right central hemisphere proband. The facial appearance of the individuals participated in the study are shown in **Figure 3**.

#### Patient 2

Patient 2 (IV-2) is 3 years and 4 months old, and he was born by normal vaginal delivery at full term without any complication. He showed a delay in speaking and walking. The patient shows hyperactivity, and a MRI examination was normal. EEG record during sleep revealed a background of predominately beta waves. Paroxysmal epileptic discharge was also noted in IV-2 the affected individuals, primarily in the right central hemisphere proband.

### Magnetic Resonance Imaging Testing

A hypointense lesion was noted in T1W and in T2W MRI images in the left frontal area, and it measured 5.4 cm by 2.10 cm. It is extra-axial to the brain and causes some mass effect on the left frontal lobe with no midline shift. The ventricle's and basal cistern's normal posterior fossa were normal. The gray-white matter junction and brain stem were normal. These findings are consistent with a cystic collection in the left frontal area.

### Whole Exome Sequencing

WES was done to identify the pathogenic mutation related to this disorder phenotype, we prepared samples and did WES using the Illumina HiSeq 2000/2500 system. We set up our sampling by following the Agilent SureSelect Target Enrichment Kit preparation guide (Capture Kit, SureSelect v.6). The libraries were sequenced utilizing the Illumina HiSeq 2000/2500 system. The variants were clarified by using various parameters, such as quality, quantity, frequency, genomic position, effect on protein, and pathogenicity. Diverse bioinformatics investigations were made to distinguish causative variant co-segregating for *NT5C2* phenotypes as an autosomal recessive manner. To use the BWA Aligner (http://bio-bwa.Sourceforge.Net/), the crude information FASTQ files were adjusted. Copy number variation, insertion, and deletion were distinguished by utilizing SAMtools (http://samtools.Sourceforge.Net/). The hg19 human reference data base was used as reference (National Center for Biotechnology Information assembly GRCh37, http://genome.Ucsc.Edu/). Furthermore, the data were plotted against the information in the dbSNP (http://www.Ncbi.Nlm.Nih.Gov/snp/) and 1000 Genomes databases (http://www.1000genomes.Org/data). The separated variants were anticipated using autosomal recessive inheritance types (homozygous or compound heterozygous state) provided for the reported history of the affected family. Because the affected patients are related, the homozygous variants were very important. We aimed at finding a novel homozygous transformation in the protein coding districts of all genes that had been related to one of the siblings, and produced one reasonable candidate in the *NT5C2* gene.

### Sanger Sequencing

After WES, targeted primers were designed for Sanger sequencing as was done previously (Naseer et al., 2018). Primer for the targeted region was designed for Sanger sequencing NT5C2F: 5'-TGATGCTTTCCCTTCTGTGA-3' NT5C2R: 5'-GCAATGTGGCATCTCTCACT-3'. As a result of Sanger sequencing, raw sequence data files were obtained in the AB1 sequence trace format. Each sequence trace file was aligned to the corresponding reference sequence using the BioEdit software packages. We searched for the observed variations in the NCBI database for SNPs. Moreover, this pathogenic variants were also sequenced in the unrelated 100 control sample from the Saudi population.

## Data Availability Statement

To maintain the anonymity of the family, the datasets in this paper will not be publicly deposited. Requests to access the datasets should be directed to MN (mimrannaseer@yahoo.com).

## Ethics Statement

The studies involving human participants were reviewed and approved by Ethical committee of CEGMR KAU approved this study. Written informed consent to participate in this study was provided by the participants' legal guardian/next of kin. Written informed consent was obtained from the individual(s), and minor(s)' legal guardian/next of kin, for the publication of any potentially identifiable images or data included in this article.

## Author Contributions

MN, PP and AC designed the experiments. AA, FB, MN conducted the expirments. PP, MN and AC analyzed the data. MN and FB wrote the manuscript.

## Acknowledgment

This project was funded by the Deanship of Scientific Research (DSR), King Abdulaziz University, Jeddah, under grant No. (DF-489-142-1441). The authors, therefore, gratefully acknowledge DSR technology and financial support.

## Conflict of Interest

The authors declare that the research was conducted in the absence of any commercial or financial relationships that could be construed as a potential conflict of interest.
